# Sustained Inattentional Blindness Does Not Always Decrease With Age

**DOI:** 10.3389/fpsyg.2018.01390

**Published:** 2018-08-29

**Authors:** Hui Zhang, Congcong Yan, Xingli Zhang, Jie Fang

**Affiliations:** ^1^Hangzhou College of Preschool Teacher Education, Zhejiang Normal University, Hangzhou, China; ^2^Key Laboratory of Behavioral Science, Institute of Psychology, Chinese Academy of Sciences, Beijing, China

**Keywords:** sustained inattentional blindness, developmental difference, primary task, motion task, difficulty level

## Abstract

Children usually miss additional information when they focus on objects or events. This common phenomenon is termed as inattentional blindness. To explore the age-related degree of this phenomenon, we applied a motion task to study the developmental difference of inattentional blindness. A group of 7-to-14-year-old children and adults participated in Experiment 1. The results showed that there was no significant developmental difference in sustained inattentional blindness. Considering that young children’s performance on the primary task was poor, we hypothesized that the difficulty of the primary task may contribute to the negative findings. Therefore, we decreased the difficulty of the primary task in Experiment 2. Still, the developmental difference in inattentional blindness rates was absent. Overall, current results implied that the ability of a person to detect an unexpected moving stimuli does not always increase with age. The age-related inattentional blindness seems highly dependent on tasks.

## Introduction

When engaged in an absorbing task, children tend to look without seeing additional stimuli. For instance, they cannot detect their parents or parents’ directions when they are watching cartoons or playing with toys; they cannot perceive a car coming toward them when they are talking excitedly about interesting things (Bornstein, 1990; Fagan and Haiken-Vasen, 1997). However, do these cases decrease with age?

The phenomenon of people failing to perceive an additional stimulus when they are engaged in a cognitively demanding task is termed as inattentional blindness (IB) (Mack and Rock, 1998; Simons and Chabris, 1999). IB happens frequently in our daily life and even contributes to some accidents in traffic or medical fields (Hannon and Richards, 2010; Hughes-Hallett et al., 2015). Therefore, a question arises as to whether some people are more likely to experience IB than others. Certain studies have focused on some individual differences of IB. For example, some researchers have investigated whether younger children are more likely to experience IB than older children (Memmert, 2006, 2014; Remington et al., 2014), and whether people who have large working memory capacities or higher fluid intelligence scores are more likely to avoid IB (Hannon and Richards, 2010; Seegmiller et al., 2011; Bredemeier and Simons, 2012; O’Shea and Fieo, 2014; Grossman et al., 2015; Kreitz et al., 2015a; Zhang et al., 2016).

To the best of our knowledge, three studies have focused on the developmental differences of IB in children. Memmert (2006) adopted the “gorilla video” paradigm to study how the children treated an unexpected moving “gorilla” when they performed a sustained attentional task. In the “gorilla video,” two groups of players dressed in different colors formed a circle together to pass the basketball. Participants needed to count the number of passes by a team of players within a given period of time. During this time, an actor dressed in a gorilla costume walked past the players and the detection of the “gorilla” was an indicator of an individual not experiencing IB. This study showed that 8-year-old children were more likely to fail than 13-year-old teenagers and adults to notice a person dressed in a gorilla suit walking among the players. Since children in only one age group were employed in this study, the developmental conclusions were limited. Therefore, Memmert (2014) conducted another supplementary study with children in different age groups. Four hundred and eighty 8-to-15-year-olds participated in his study, and it was found that 8-to-10-year-olds’ IB rate was significantly higher than 11-to-15-year-olds’. There was no significant difference between 8-to-10-year-old and 11-to-15-year-old children.

Remington et al. (2014) applied the “line-length judgment task” to investigate the developmental differences of IB in 7-to-14-year-old children. In the “line-length judgment task,” participants should determine which line on a fast-emerging cross was longer. In one trial, an unexpected object appeared in one quadrant of the cross. Participants were asked if they detected something other than the cross, and they also needed to explain the shape and position of the object. Remington et al. (2014) adjusted the perceptual load of the primary task by changing the line-length difference and set the unexpected stimuli in the central or peripheral areas. The results showed that children’s detection of an unexpected stimulus increased with age ranging from 7 to 14 years, but the turning point of development changed under different parameters.

These former studies on the developmental differences in IB show that IB develops by phase and the turning point changes according to the paradigm and the detailed parameters of the paradigm. According to Lavie and Tsal (1994) perceptual load model, whether people can detect the task-independent stimuli depends critically on whether they have surplus cognitive resources after focusing on task-related stimuli. It can be summarized that the mechanism of limited resources in people can account for some incidence of IB. Younger children are more likely to experience IB than older children, and this may be due to the lack of cognitive resources to process task-irrelevant stimuli.

However, there is evidence that IB occurs even when cognitive resources are abundant. Participants might be blind to non-target stimuli if the task instructions did not specifically direct them to pay attention to those stimuli, even if they had sufficient perceptual capacity (Eitam et al., 2013). It can be interpreted that people who insisted more on the instructions would be more likely to experience IB. A study conducted by Conway et al. (2001) showed that the participants who have lower self-control levels are more likely to report irrelevant messages. It implied that the people who could control themselves well would be more likely to insist on the instructions and experience IB. In addition, along with the age increase, children can maintain the activation of relevant stimuli and better inhibit irrelevant stimuli (Huizinga et al., 2006; Conklin et al., 2007). If the impact of the executive function exceeds the limited resource, there is another possibility that the IB rates would increase with age in childhood.

In brief, the developmental differences of IB can reveal the cognitive mechanism of IB to some extent; i.e., age-related-IB is better dominated by the limited resource mechanism or the executive function mechanism. Decreasing IB rates with age support the limited resource mechanism (Memmert, 2006, 2014; Remington et al., 2014). In other words, if the IB rates increase with age, the executive function mechanism will overtake the age difference in IB (Conway et al., 2001). Moreover, if there is no significant age difference in IB, it is probable that these two mechanisms of opposite effects may play roles simultaneously in the developmental differences of IB, and hence there is no significant difference of IB rates in different age groups. If the last hypothesis is verified, age-related IB should be considered more systematically from other individual characteristics.

As we know, the ecological validity of a motion task (sustained IB paradigm: primary task and unexpected stimuli are both dynamic) is better than the “line-length judgment task” (static IB paradigm: primary task and unexpected stimuli are both static). In the “line-length judgment task,” the primary task and the unexpected stimuli both occurred only within hundreds of milliseconds (Mack and Rock, 1998). They are rare in daily life. Meanwhile, the structure of the motion task is stronger than that of the “gorilla video” paradigm. The moving speed, the size of the letters, and the background of the screen are stationary in the motion task. However, as in a sustained IB paradigm, the size of the players and the speed of passing the basketball are not stationary in the “gorilla video” paradigm, and sometimes the screen background changed (Simons and Chabris, 1999). From overall considerations, we selected the motion task to investigate the developmental difference of children and adults in the current study.

Given that high attention is needed in the motion task, children under 7 years of age are not well-focused (Betts et al., 2006), and the IB of 14-year-old children is relatively stable (Memmert, 2006). For these reasons, we aimed to investigate 7-to-14-year-old children as well as adults in the current study.

## Experiment 1

### Methods

#### Participants

Two hundred and ten people participated in the present study. They were recruited from two schools in Beijing and Hangzhou, China. We removed from the analysis the participants whose accuracy of the counting task in the inattentional trial was less than two standard deviations of the average value in their age group (total 10 participants). Seven other participants were removed from the analysis because of computer errors. After exclusions, the experimental age groups consisted of the following participants (N, mean age ± SD): 7–8-year-olds (40, 7.53 ± 0.60), 9–10-year-olds (35, 9.60 ± 0.55), 11–12-year-olds (29, 11.60 ± 0.35), 13–14-year-olds (44, 13.00 ± 0.55), and adults (45, 18.71 ± 0.76).

All the participants had normal or corrected normal vision. The participants did not have any clinical or subclinical conditions that could affect their performance and they did not take any medication that could affect their performance. Before participation, parents and children or adult participants signed the informed consent forms. The experiment was approved by the Ethics Committee of the Institute of Psychology, Chinese Academy of Sciences.

### Materials

**FIGURE 1:**
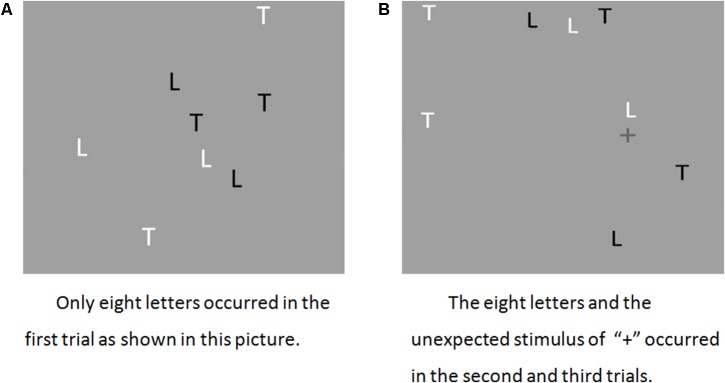
Panel **(A)** shows only eight letters occurred in the first trail. **(B)** The eight letters and the unexpected stimulus of “+” occurred in the second and third trails.

The IB task used in this study was similar to the typical motion task (Most et al., 2001; see **Figure 1**). The background color of every animated segment was light gray (15.9 cd/m^2^) and the expected items were four white (35.2 cd/m^2^) shapes (two Ls, 1.4° × 0.7° and two Ts, 1.1° × 1.4°) and four black (3.6 cd/m^2^) shapes (two Ls, 1.4° × 0.7° and two Ts, 1.1° × 1.4°) that moved independently along straight paths, occasionally bouncing off the display edges. Participants were instructed to count the number of times the four black shapes bounced off the edges during one trial (18 s) and to ignore the movements of the four white shapes. The total bouncing time was 33–34 s. In total, the participants completed three trials. The first trial was a practice run without any unexpected stimulus. The second trial was an inattentional trial. An unexpected stimulus, a dark gray plus sign (“+”) (7.3 cd/m^2^, 1.2° × 1.3°), moved along the central horizontal line from left to right for approximately 6–12 s.

After completing the counting task, the participants were asked a series of questions about the unexpected stimulus (see **Supplementary Material A** for the exact wording of all the questions). When a participant reported that he had noticed an additional stimulus and could select the correct shape and location of the stimulus that occurred during the trial, that participant was placed in the “non-inattentional blindness” (NIB) category; others were placed in the “IB” category. The third trial was a divided-attentional trial that was identical to the preceding inattentional trial except that, before the trial, participants were instructed that an additional stimulus would occur and that they should divide their attention to the additional stimulus while performing the counting task. If, after this trial, the participants reported that they had noticed the additional stimulus and could select the right shape and the path of the stimulus, those participants were placed in the “non-divided blindness” (NDB) category; others were placed in the “divided blindness” (DB) category.

### Procedure

For children, these experiments were conducted in groups, with 5–6 participants per group and they were grouped according to their age. Participants were tested in a meeting room (with an area of approximately 300 m^2^) with six laptops placed in six different locations. The IB tasks were presented on the laptops, which had 14.1-inch monitors, with a refresh rate of 60 Hz and a screen resolution of 1,024 × 768 pixels. Participants were seated at a distance of approximately 57 cm from the screen. Three experimenters were present in these experiments. One experimenter explained the needs of the task outside the lab. Participants were allowed to start the formal experiment only if they could correctly answer the questions concerning the actions involved in the test (see **Supplementary Material B** for the exact wording of the experiment request and all the questions the experimenter asked). It took each participant approximately 8–10 min to complete the tasks. All the participants completed the IB task in one morning to avoid the dissemination of experimental information among the participants who had not completed the experiment.

For adults, considering that most of them could understand the experiment requests well, the experiments were conducted in two groups in the computer classroom.

### Results

The accuracy of the primary task, the IB rates, and the DB rates of different age groups are shown in **Table 1**. We compared the IB rates of different age groups by using the chi-square test. The results showed that there was no significant developmental difference of IB rates in 7-to-14-year-old children and adults [*χ*^2^ (4) = 1.222, *p* = 0.874]. Additionally, we also compared the DB rates among these groups by performing chi-square tests. The results showed that there was no significant developmental difference of DB rates in 7-to-14-year-old children and adults (*χ*^2^ (4) = 5.950, *p* = 0.203).

**Table 1:** IB rates and accuracy in primary task of different age groups in Experiment 1.

	ACC_1_	ACC_2_	IB rate	DB rate
7–8 years	0.733 (0.201)	0.733 (0.206)	60.0%	40.0%
9–10 years	0.822 (0.150)	0.786 (0.204)	65.7%	51.4%
11–12 years	0.815 (0.169)	0.781 (0.243)	69.0%	31.0%
13–14 years	0.906 (0.074)	0.821 (0.207)	59.1%	34.1%
Adults	0.888 (0.084)	0.891 (0.083)	66.7%	26.7%


*Notes*: $ACC=\frac{\left|correctcounting-parcipantscounting\right|}{correctcounting},$

$IB\;rate=\frac{IB}{IB+NIB},$

$DB\;rate=\frac{DB}{DB+NDB}$

We compared the accuracy of the primary task among these age groups (accuracy in the inattentional trial was recorded as ACC_1_; accuracy in the divided trial was recorded as ACC_2_) by using ANOVA. The analysis revealed a significant increase in ACC_1_ and ACC_2_ with age: ACC_1_, *F*(4,188) = 9.984, *p* < 0.000, partial η*^2^* = 0.175; ACC_2_, *F*(4,181) = 3.995, *p* = 0.004, partial η*^2^* = 0.081.

Meanwhile, we also investigated the relationship between IB and the accuracy of the primary task by using a *T*-test. The ACC_1_ of IBs and NIBs are shown in **Table 2** and the ACC_2_ of IBs and NIBs are shown in **Table 3**. The results showed that there was no significant difference between IBs and NIBs in terms of the accuracy of the primary task in every age group. However, the results of the paired sample *T*-test showed that ACC_1_ was significantly higher than ACC_2_ for 7-to-14-year-olds and adults [*t*(186) = 2.398, *p* = 0.017].

**Table 2:** ACC_1_ of IBs and NIBs in Experiment 1.

ACC_1_	IB	NIB	*t*	*p*
7–8 years	0.722 (0.206)	0.750 (0.200)	0.431	0.669
9–10 years	0.850 (0.130)	0.769 (0.176)	-1.548	0.131
11–12 years	0.848 (0.154)	0.741 (0.186)	-1.630	0.115
13–14 years	0.907 (0.073)	0.906 (0.077)	0.060	0.953
Adults	0.882 (0.084)	0.898 (0.079)	0.602	0.550


**Table 3:** ACC_2_ of IBs and NIBs in Experiment 1.

ACC_2_	IB	NIB	*t*	*p*
7–8 years	0.756 (0.203)	0.698 (0.212)	-0.220	0.826
9–10 years	0.835 (0.142)	0.694 (0.269)	-2.006	0.053
11–12 years	0.784 (0.254)	0.777 (0.232)	-0.062	0.951
13–14 years	0.862 (0.112)	0.759 (0.291)	-1.348	0.194
Adults	0.893 (0.074)	0.886 (0.100)	-0.266	0.792


### Discussion

More than half of the participants suffered from IB during this experiment. This result confirms that IB is indeed a common phenomenon (Simons and Chabris, 1999) not only in childhood but also in adulthood. However, the current results showed that even if adults and older children could count the crash times more accurately, the detection rate of the unexpected dynamic object was not higher than that for the younger children. In addition, there was no significant difference between the results of IB and the performance of the primary tasks. This is also consistent with the results of previous studies (Simons and Jensen, 2009; Bredemeier and Simons, 2012); i.e., whether or not an unexpected stimulus was detected is not directly related to the performance of the primary task.

Children’s cognitive abilities develop simultaneously with age. With the increase in age, children can process information faster (Kail and Salthouse, 1994); their attention abilities are better (Rebok et al., 1997); their intelligence is higher (Arthur and Day, 1994; Lynn et al., 2004); and their working memory capacities are larger (Hambrick and Engle, 2002; Rhodes and Kelley, 2005). These evidences indicate that children’s perceptual capacities develop during childhood. Considering the mechanism of limited resources, it seems that adults and older children would be more likely to process the unexpected stimuli because their perceptual capacities are higher. However, the present results showed that there was no significant developmental difference of IB rates between children aged 7–14 years and adults. This finding is inconsistent with the previous findings that children’s IB rates decreased with age (Memmert, 2006, 2014; Remington et al., 2014).

The negative findings on the developmental difference of IB need to be treated with caution. In fact, Neisser’s initial study of children’s selective attention suggested that children have a higher incidence of detecting unexpected stimuli compared with adults (Neisser, 1979). It means that children’s IB rate is lower than that of adults. However, Neisser was critical of this finding. Considering that some children had not successfully completed the primary task, Neisser supposed that the finding that children shift to unexpected stimuli should be attributed to their inability to remain well-focused on the primary moving task. Neisser explained that it is logical to hypothesize that younger children are not less likely to detect unexpected stimuli than older children and adults, probably because they cannot focus on the main task.

Indeed, the performance of the primary task of children aged 7–8 years was not satisfactory. Their mean accuracy of the primary task was worse than 80%. If we apply this criterion for adult participants, only the participants whose accuracy reaches 80% can be considered as “on task” in the primary task (Seegmiller et al., 2011); i.e., most of the 7–8-year-old children were “off task” in the primary task. Therefore, the fact that children could not remain focused on the primary task may account for the negative findings of Experiment 1. To explore the effect of the primary task, we decreased the difficulty of the primary task in Experiment 2 so that most children can be “on task” in the primary task.

## Experiment 2

### Methods

#### Participants

One hundred and ninety-one people participated in the present study. They were recruited from Hangzhou and Shaoxing, China. From the analysis, we removed eight participants whose accuracy in the counting task in the inattentional trial was less than two standard deviations of the average value in their age group. Seven participants were removed from the analysis because of computer errors. After exclusions, the experimental age groups consisted of the following participants (N, mean age ± SD): 7–8-year-olds (32, 7.72 ± 0.52), 9–10-year-olds (31, 9.61 ± 0.50), 11–12-year-olds (36, 11.73 ± 0.45), 13–14-year-olds (42, 13.24 ± 0.43), and adults (39, 18.24 ± 0.49).

All the participants had normal or corrected normal vision. The participants did not have any clinical or subclinical conditions that could affect their performance and they did not take any medication that could affect their performance. Before participation, parents and children or adult participants signed informed consent forms. The experiment was approved by the Ethics Committee of the Institute of Psychology, Chinese Academy of Sciences.

### Materials

The IB task was identical to that of Experiment 1 except that the target moves more slowly and the crash time of the primary task is less than that of Experiment 1. The total bouncing time was 22–23 s.

### Procedure

These experiments were administered in a manner similar to Experiment 1, except that children were tested in computer rooms using only five computers. The seat arrangement was fully considered to avoid the interference from each other. The adult experiments were administered in the same way as Experiment 1.

### Results

The accuracy of the primary task as well as the IB rates and the DB rates of different age groups are presented in **Table 4**. We compared the IB rates of different age groups by using the chi-square test. The results showed no significant difference in the IB rates between 7-to-14-year-old children and adults [*χ*^2^(4) = 3.944, *p* = 0.414]. Additionally, we compared the DB rates of these groups by performing chi-square tests in the current experiment. The results showed that the DB rates significantly decreased with age in 7-to-14-year-old children and adults [*χ*^2^(4) = 22.938, *p* < 0.000].

**Table 4:** IB rates and accuracy in primary task of different age groups in Experiment 2.

	ACC_1_	ACC_2_	IB rate	DB rate
7–8 years	0.803 (0.098)	0.736 (0.298)	71.9%	56.3%
9–10 years	0.826 (0.198)	0.777 (0.190)	83.9%	32.3%
11–12 years	0.906 (0.162)	0.808 (0.295)	76.7%	23.3%
13–14 years	0.909 (0.063)	0.907 (0.087)	88.1%	14.3%
Adults	0.916 (0.074)	0.916 (0.073)	75.6%	12.2%


In addition, we investigated the relationship between IB and the accuracy of the primary task by performing a *T*-test. The ACC_1_ of IBs and NIBs are listed in **Table 5**, and the ACC_2_ of IBs and NIBs are listed in **Table 6**. The *T*-test results showed that no significant difference exists between IBs and NIBs in terms of the accuracy of the primary task in every age group. However, the results of the paired sample *T*-test showed that ACC_1_ was significantly higher than ACC_2_ for 7-to-14-year-olds and adults [*t*(175) = 2.404, *p* = 0.017].

**Table 5:** ACC_1_ of IBs and NIBs in Experiment 2.

ACC_1_	IB	NIB	*t*	*p*
7–8 years	0.795 (0.098)	0.823 (0.103)	0.738	0.466
9–10 years	0.843 (0.134)	0.737 (0.413)	-0.569	0.598
11–12 years	0.923 (0.143)	0.851 (0.219)	-1.033	0.311
13–14 years	0.913 (0.062)	0.882 (0.069)	-1.037	0.306
Adults	0.915 (0.075)	0.918 (0.074)	0.119	0.906


**Table 6:** ACC_2_ of IBs and NIBs in Experiment 2.

ACC_2_	IB	NIB	*t*	*p*
7–8 years	0.682 (0.333)	0.874 (0.093)	1.687	0.102
9–10 years	0.764 (0.201)	0.845 (0.105)	0.876	0.388
11–12 years	0.771 (0.328)	0.929 (0.044)	2.238	0.035
13–14 years	0.909 (0.091)	0.891 (0.052)	-0.435	0.666
Adults	0.915 (0.078)	0.918 (0.060)	0.120	0.905


Moreover, we compared the IB rates under high load (Experiment 1) and low load (Experiment 2) conditions in different age groups by using the chi-square test. We found that the IB rates under low load was significantly higher than that under high load in 13–14-year-old children [*χ*^2^(1) = 9.227, *p* = 0.002]. The accuracy of the primary task both under high and low loads were also compared by using the *T*-test in different age groups. The results showed that ACC_1_ under low load was higher than that under high load in 7–8-year-old children [*t*(70) = 1.918, *p* = 0.06] and 11–12-year-old children [*t*(57) = 2.109, *p* = 0.039].

### Discussion

The current results did not confirm the hypothesis that the difficulty of the primary task may be attributed to the negative findings of the developmental differences in IB rates. We even decreased the difficulty of the primary task and made most of the participants “on task” in the primary task; yet the children detecting the unexpected stimuli did not increase with age. It would be an exaggeration to say that there is no significant developmental difference of sustained IB rates, but it is logical to indicate that the detection of the unexpected moving object by children did not increase with age in the current two cases.

After the crash times of the primary task were reduced in the present experiment, the participants could perform better in the primary task than in Experiment 1. However, an interesting result showed that the participants’ detection of the unexpected moving object decreased when the difficulty of the primary task decreased in some age groups. This result was inconsistent with the previous findings that the IB rates would decrease if the difficulty of the primary task is decreased (Simon et al., 1999; Todd et al., 2005; Cartwright-Finch and Lavie, 2007; Calvillo and Jackson, 2013). However, de Fockert and Bremner’s (2011) study showed that decreasing the difficulty of the primary task might also increase the IB rate, if stimulus detection is competing for attention with a concurrent visual task. It means that when the crash times decreased, the primary task would consume moderate attentional resource, and then participants could maintain the attention on the current task and inhibit task-irrelevant stimuli better. Therefore, when the primary task was easier, it was more possible for participants to experience IB.

However, we found that there was developmental difference of DB rates in the current experiment. Adults and older children better detected the additional stimuli when they were instructed to divide attention to additional stimuli. This implied that the developmental differences of the DB rates were more sensitive to the primary task.

## General Discussion

Combined together, the results of these two experiments indicate that there are no developmental differences of sustained IB rates in the current two motion tasks. These results are consistent with the notion that the individual differences in detecting the unexpected object depends on the detailed experimental settings (Kreitz et al., 2015a). However, in the two experiments, the performance of participants in the primary task revealed that attention capacity developed with age (Paus, 1989; Klimkeit et al., 2004; Betts et al., 2006).

It must be mentioned that our results are inconsistent with the findings that children’s IB rates decrease with age (Memmert, 2006, 2014; Remington et al., 2014). Compared with sustained IB, static IB is more sensitive to the cognitive resources. Kreitz et al. (2015a) explored the relationship between IB and cognitive abilities. They found that working memory capacity can predict attention only in central static IB tasks, and there is no cognitive measure to predict sustained IB. Therefore, it was more likely to show significant developmental differences in static IB (Remington et al., 2014). In addition, Memmert’s (2006, 2014) research applied the “gorilla video” task, a sustained IB paradigm, and found significant developmental differences of IB. These results should be attributed to the different paradigm of sustained IB compared with the current research. Therefore, compared with the “gorilla video” task, the motion task has more influential factors when it is applied to test IB (Seegmiller et al., 2011; Bredemeier and Simons, 2012; Kreitz et al., 2015a). Our hypothesis here that the limited resource and executive function mechanisms affect the developmental differences of IB simultaneously in 7-to-14-year-olds and adults reflects that the IB rates do not differ with the age of participants, but this opinion should be tested in substantive experiments.

A growing literature has sought to pay attention to the individual differences of IB. Researchers expected to find some predictors of IB, but most of them have not found positive results. Kreitz et al. (2015b) investigated the relevance of IB and personality traits. They found that openness was a predictor of IB. However, other studies did not show the correlation between IB and personality traits, such as emotional distress, anxiety, worry, depression, schizotypy, and achievement motivation (Bredemeier et al., 2014). Moreover, the series of studies on the topic of IB and working memory do not appear closely related between these two cognitive performances However, this phenomenon may be influenced by multiple factors which may affect IB when exploring the individual difference of IB. Hence, when we investigate the individual differences of IB, it seems necessary to examine the characteristics of all aspects at the same time, such as age, personality, and cognitive ability.

Even though no significant developmental differences of the IB rates exist in the two different conditions, it is interesting to find that adults and older children are more likely to detect moving objects under the divided attentional condition compared with the younger children with a low difficulty level of the primary task. This implies that children’s divided attention develops with age in some conditions, consistent with other serious evidences (Paus, 1989; Klimkeit et al., 2004; Betts et al., 2006). Additionally, both the experiments show that participants’ performance in the primary task would reduce in a divided attentional trial compared with an inattentional trial. It means that whether or not a participant detects an object, the presence of the object automatically captures the participant’s attention, consistent with the evidence of attention capture (Theeuwes, 1994).

Actually, the negative findings should be treated cautiously. There are some limitations that we should admit. First, the difficulty of the primary task should be further adjusted so as to systematically investigate the effect of the primary task in the developmental differences of IB. Another possibility is that no developmental differences on IB rates should be attributed to the high difficulty of the primary tasks even in Experiment 2. However, if nearly every participant could totally respond correctly to the primary task, will the developmental differences of IB rates occur? It is a question we should deal with in a future study. In addition, as there was still a part of the participants who could not detect the unexpected stimuli in the divided attentional trial, a full attentional trial should be added to test whether every participant could detect the unexpected stimuli in the full attentional condition. Moreover, individual personality traits and cognitive abilities should be systematically examined in a task package in our future studies. This can help us learn more about the roles of these factors in the development of IB so as to further analyze the cognitive mechanism of the developmental differences in IB. In general, the present study indicated that no significant differences exist in IB rates between 7-to-14-year-olds and adults in the motion task, and the IB–age-difference relationship was strongly dependent on the tasks. In other words, age alone is not a stable predictor for IB.

## Ethics Statement

This study was carried out in accordance with the recommendations of Ethical rules of human participants, the Ethics Committee from the Institute of Psychology, Chinese Academy of Sciences with written informed consent from all subjects. All subjects and their parents gave written informed consent in accordance with the Declaration of Helsinki. The protocol was approved by Ethics Committee from the Institute of Psychology, Chinese Academy of Sciences.

## Author Contributions

HZ designed the paper, collected the data, and wrote the paper. CY collected the data and wrote the paper. XZ designed the paper. JF collected the data.

## Conflict of Interest Statement

The authors declare that the research was conducted in the absence of any commercial or financial relationships that could be construed as a potential conflict of interest.

## References

[B1] Arthur W. Day D. V. (1994). Development of a short form for the raven advanced progressive matrices test. *Educ. Psychol. Meas.* 54 394–403. 10.1177/0013164494054002013

[B2] Beanland V. Chan E. H. (2016). The relationship between sustained inattentional blindness and working memory capacity. *Atten. Percept. Psychophys.* 78 808–817. 10.3758/s13414-015-1027-x 26754810

[B3] Betts J. McKay J. Maruff P. Anderson V. (2006). The development of sustained attention in children: the effect of age and task load. *Child Neuropsychol.* 12 205–221. 10.1080/09297040500488522 16837396

[B4] Bornstein M. H. (1990). Attention in infancy and the prediction of cognitive capacities in childhood. *Adv. Psychol.* 69 3–19. 10.1016/S0166-4115(08)60448-3 2688119

[B5] Bredemeier K. Hur J. Berenbaum H. Heller W. Simons D. J. (2014). Individual differences in emotional distress and susceptibility to inattentional blindness. *Psychol. Conscious. Theory Res. Pract.* 1 370–386. 10.1037/cns0000032

[B6] Bredemeier K. Simons D. J. (2012). Working memory and inattentional blindness. *Psychon. Bull. Rev.* 19 239–244. 10.3758/s13423-011-0204-8 22222359

[B7] Calvillo D. P. Jackson R. E. (2013). Animacy, perceptual load, and inattentional blindness. *Psychon. Bull. Rev.* 21 670–675. 10.3758/s13423-013-0543-8 24197657

[B8] Cartwright-Finch U. Lavie N. (2007). The role of perceptual load in inattentional blindness. *Cognition* 102 321–340. 10.1016/j.cognition.2006.01.002 16480973

[B9] Conklin H. M. Luciana M. Hooper C. J. Yarger R. S. (2007). Working memory performance in typically developing children and adolescents: behavioral evidence of protracted frontal lobe development. *Dev. Neuropsychol.* 31 103–128. 10.1080/87565640709336889 17305440

[B10] Conway A. R. A. Cowan N. Bunting M. F. (2001). The cocktail party phenomenon revisited: the importance of working memory capacity. *Psychon. Bull. Rev.* 8 331–335. 10.3758/BF03196169 11495122

[B11] de Fockert J. W. Bremner A. J. (2011). Release of inattentional blindness by high working memory load: elucidating the relationship between working memory and selective attention. *Cognition* 121 400–408. 10.1016/j.cognition.2011.08.016 21937032

[B12] Eitam B. Yeshurun Y. Hassan K. (2013). Blinded by irrelevance: pure irrelevance induced “blindness”. *J. Exp. Psychol.* 39 611–615. 10.1037/a0032269 23506113

[B13] Fagan J. F. Haiken-Vasen J. (1997). “Selective attention to novelty as a measure of information processing across the lifespan,” in *Attention, Development, and Sustained Attention and Injury 81 Psychopathology* eds Burrack J. A. Enns J. T. (New York, NY: Guilford Press) 55–73.

[B14] Grossman E. S. Hoffman Y. S. G. Berger I. Zivotofsky A. Z. (2015). Beating their chests: university students with ADHD demonstrate greater attentional abilities on an inattentional blindness paradigm. *Neuropsychology* 29 882–887. 10.1037/neu0000189 25730730

[B15] Hambrick D. Z. Engle R. W. (2002). Effects of domain knowledge, working memory capacity, and age on cognitive performance: an investigation of the knowledge-is-power hypothesis. *Cogn. Psychol.* 44 339–387. 10.1006/cogp.2001.0769 12018938

[B16] Hannon E. M. Richards A. (2010). Is inattentional blindness related to individual differences in visual working memory capacity or executive control functioning? *Perception* 39 309–319. 10.1068/p6379 20465168

[B17] Hughes-Hallett A. Mayer E. K. Marcus H. J. Pratt P. Mason S. Darzi A. W. (2015). Inattention blindness in surgery. *Endosc. Surg.* 29 3184–3189. 10.1007/s00464-014-4051-3 25582962

[B18] Huizinga M. Dolan C. V. Van D. M. (2006). Age-related change in executive function: developmental trends and a latent variable analysis. *Neuropsychologia* 44 2017–2036. 10.1016/j.neuropsychologia.2006.01.010 16527316

[B19] Kail R. Salthouse T. A. (1994). Processing speed as a mental capacity. *Acta Psychol.* 86 199–225. 10.1016/0001-6918(94)90003-5 7976467

[B20] Klimkeit E. I. Mattingley J. B. Sheppard D. M. Farrow M. Bradshaw J. L. (2004). Examining the development of attention and executive functions in children with a novel paradigm. *Child Neuropsychol.* 10 201–211. 10.1080/09297040409609811 15590499

[B21] Kreitz C. Furley P. Memmert D. Simons D. J. (2015a). Inattentional blindness and individual differences in cognitive abilities. *PLoS One* 10:e0134675. 10.1371/journal.pone.0134675 26258545 PMC4530948

[B22] Kreitz C. Schnuerch R. Gibbons H. Memmert D. (2015b). Some see it, some don’t: exploring the relation between inattentional blindness and personality factors. *PLoS One* 10:e0128158. 10.1371/journal.pone.0128158 26011567 PMC4443971

[B23] Kreitz C. Furley P. Simons D. J. Memmert D. (2016). Does working memory capacity predict cross-modally induced failures of awareness? *Conscious. Cogn.* 39 18–27. 10.1016/j.concog.2015.11.010 26658847

[B24] Lavie N. Tsal Y. (1994). Perceptual load as a major determinant of the locus of selection in visual attention. *Percept. Psychophys.* 56 183–197. 10.3758/BF03213897 7971119

[B25] Lynn R. Allik J. Irwing P. (2004). Sex differences on three factors identified in Raven’s Standard Progressive Matrices. *Intelligence* 32 411–424. 10.1016/j.intell.2004.06.007 11327166

[B26] Mack A. Rock I. (1998). *Inattentional Blindness.* Cambridge, MA: MIT Press.

[B27] Memmert D. (2006). The effects of eye movements, age, and expertise on inattentional blindness. *Conscious. Cogn.* 15 620–627. 10.1016/j.concog.2006.01.001 16487725

[B28] Memmert D. (2014). Inattentional blindness to unexpected events in 8-15-year-olds. *Cogn. Dev.* 32 103–109. 10.1016/j.cogdev.2014.09.002

[B29] Most S. B. Simons D. J. Scholl B. J. Jimenez R. Clifford E. Chabris C. F. (2001). How not to be seen: the contribution of similarity and selective ignoring to sustained inattentional blindness. *Psychol. Sci.* 12 9–17. 10.1111/1467-9280.00303 11294235

[B30] Neisser U. (1979). “The control of information pickup in selective looking,” in *Perception and its Development: A Tribute to Eleanor J. Gibson* ed. Pick A. D. (Hillsdale, NJ: Erlbaum) 201–219.

[B31] O’Shea D. M. Fieo R. A. (2014). Individual differences in fluid intelligence predicts inattentional blindness in a sample of older adults: a preliminary study. *Psychol. Res.* 79 570–578. 10.1007/s00426-014-0594-0 25001000

[B32] Paus T. (1989). The development of sustained attention in children might be related to the maturation of frontal cortical functions. *Acta Neurobiol. Exp.* 49 51–55. 2718790

[B33] Rebok G. W. Smith C. B. Pascualvaca D. M. Mirsky A. F. Anthony B. J. Kellam S. G. (1997). Developmental changes inattentional performance in urban children from eight to thirteen years. *Child Neuropsychol.* 3 28–46. 10.1080/09297049708401366

[B34] Remington A. Cartwright-Finch U. Lavie N. (2014). I can see clearly now: the effects of age and perceptual load on inattentional blindness. *Front. Hum. Neurosci.* 8:229. 10.3389/fnhum.2014.00229 24795596 PMC4005968

[B35] Rhodes M. G. Kelley C. M. (2005). Executive processes, memory accuracy, and memory monitoring: an aging and individual difference analysis. *J. Mem. Lang.* 52 578–594. 10.1016/j.jml.2005.01.014

[B36] Seegmiller J. K. Watson J. M. Strayer D. L. (2011). Individual differences in susceptibility to inattentional blindness. *J. Exp. Psychol. Learn. Mem. Cogn.* 37 785–791. 10.1037/a0022474 21299325

[B37] Simons D. J. Chabris C. F. (1999). Gorillas in our midst: sustained inattentional blindness for dynamic events. *Perception* 28 1059–1074. 10.1068/p2952 10694957

[B38] Simons D. J. Jensen M. S. (2009). The effects of individual differences and task difficulty on inattentional blindness. *Psychon. Bull. Rev.* 16 398–403. 10.3758/PBR.16.2.398 19293113

[B39] Theeuwes J. (1994). Stimulus-driven capture and attentional set: selective search for color and visual abrupt onsets. *J. Exp. Psychol.* 20 799–806. 10.1037/0096-1523.20.4.799 8083635

[B40] Todd J. J. Fougnie D. Marois R. (2005). Visual short-term memory load suppresses temporo-parietal junction activity and induces inattentional blindness. *Psychol. Sci.* 16 965–972. 10.1111/j.1467-9280.2005.01645.x 16313661

[B41] Zhang H. Zhang X. He Y. Shi J. (2016). Inattentional blindness in 9-to 10-year-old intellectually gifted children. *Gift. Child Q.* 60 287–295. 10.1177/0016986216657158

