# Supplementary material for: Sustained Inattentional Blindness Does Not Always Decrease With Age
Source: Front Psychol. 2018 Aug 29;9:1390. doi: 10.3389/fpsyg.2018.01390 (PMC6124514; doi:10.3389/fpsyg.2018.01390)
Supplement: Supplementary file 1 [file Presentation_1.PDF]

Supplementary Material A

Questionnaire on IB

1. Other than the 8 letters, did you see any additional stimulus on the screen?

   If so, please enter "1;" otherwise, enter "2."

2. Which figure represents the additional stimulus? Please input the number corresponding to the additional stimulus.

   1. = 2. + 3. - 4. # 5. % 6. ?

3. What path did the additional stimulus take on the screen? Please input the corresponding number.

   1. From left to right 2. From right to left

   3. From top to bottom 4. From bottom to top
