# Supplementary material for: Sustained Inattentional Blindness Does Not Always Decrease With Age
Source: Front Psychol. 2018 Aug 29;9:1390. doi: 10.3389/fpsyg.2018.01390 (PMC6124514; doi:10.3389/fpsyg.2018.01390)
Supplement: Supplementary file 2 [file Presentation_2.PDF]

Supplementary Material B

Instructions

Next, we will have a test for attention. You should listen to my introduction of the test carefully first and then complete the test in the room. It is a counting test. When you begin with the task, you will find that there are four black letters and four white letters in the screen. These letters are moving independently along the straight paths, occasionally bouncing off the display edges. You should only focus on the four black letters and count how many times they bounced on the edge of the screen. Just like this movie (Here, we show the movie of the first trial). So lets begin to count how many times the black letters bounced on the edge from now on (We begin to count the bounce times with the children for 5-6 seconds together). After that, we ask the children three questions as below.

1. What color of the letters should we pay attention to? (Black)
2. What edges did we mention in the task? (The edge of the screen)
3. If the three black letters bounced on the edges at the same time, how many times should we count in this bouncing? (Three)

If every child can answer these questions correctly, we will let them enter in the test room and begin the test.
